# Evaluation of active ingredients and larvicidal activity of clove and cinnamon essential oils against *Anopheles gambiae* (*sensu lato*)

**DOI:** 10.1186/s13071-017-2355-6

**Published:** 2017-09-06

**Authors:** Adelina Thomas, Humphrey D. Mazigo, Alphaxard Manjurano, Domenica Morona, Eliningaya J. Kweka

**Affiliations:** 1 School of Pharmacy, Catholic University of Health and Allied Sciences, P.O. Box 1464, Mwanza, Tanzania; 2 Department of Medical Parasitology and Entomology, School of Medicine, Catholic University of Health and Allied Sciences, P.O. Box 1464, Mwanza, Tanzania; 3 National Institute for Medical Research, Mwanza Research Centre, Mwanza, Tanzania; 4 Division of Livestock and Human Diseases Vector Control, Mosquito Section, Tropical Pesticides Research Institute, P.O. Box 3024, Arusha, Tanzania

**Keywords:** *Anopheles gambiae*, Larvicides, Mortality, Essential oil, Clove, Cinnamon

## Abstract

**Background:**

Mosquitoes are well-known vectors of many diseases including malaria and lymphatic filariasis. Uses of synthetic insecticides are associated with high toxicity, resistance, environmental pollution and limited alternative, effective synthetic insecticides. This study was undertaken to evaluate the larvicidal efficacy of clove and cinnamon essential oils against laboratory *Anopheles gambiae* (*sensu stricto*) and wild *An. arabiensis* larvae.

**Methods:**

The standard WHO guideline for larvicides evaluation was used, and the GC-MS machine was used for active compounds percentage composition analysis and structures identification. Probit regression analysis was used for LC_50_ and LC_95_ calculations while a t-test was used to test for significant differences between laboratory-reared and wild larvae populations in each concentration of plant extract.

**Results:**

Mortality effect of clove and cinnamon essential oils against wild and laboratory-reared larvae had variations indicated by their LC_50_ and LC_95_ values. The mortality at different concentrations of cinnamon and clove post-exposure for wild and laboratory-reared larvae were dosage-dependent and were higher for cinnamon than for clove essential oils. The mortality effect following exposure to a blend of the two essential oils was higher for blends containing a greater proportion of cinnamon oil. In the chemical analysis of the active ingredients of cinnamon essential oil, the main chemical content was Eugenol, and the rarest was β-Linalool while for clove essential oil, the main chemical content was Eugenol and the rarest was Bicyclo.

**Conclusion:**

The essential oils showed a larvicidal effect which was concentration-dependent for both laboratory and wild collected larvae. The active ingredient compositions triggered different responses in mortality. Further research in small-scale should be conducted with concentrated extracted compounds.

## Background

Vector-borne diseases remain a major public health concern in tropical areas [1]. Poor drainage systems in urban areas, especially during rainy seasons, and irrigation ditches in farmland provide abundant mosquito breeding places [1–3]. Tanzania spends a low share of its gross domestic product (7.2%) on health, with a meagre public expenditure of 39% for health costs [4]. Vector-borne diseases affecting people in Tanzania are malaria, filariasis, dengue, chikungunya and other arboviruses [5]. Malaria remains the deadliest vector-borne disease despite long-term control efforts [6]. Among the mosquito-borne vectors, *Ae. aegypti* (L.), a vector of dengue fever, is prevalent in the tropics and sub-tropics and is closely associated with human habitats outside its native range of Africa [7]. *Culex pipiens pallens* (Coquillett) is a vector of the western Nile virus and is distributed throughout Africa, the Middle East and Asia [8]. The complex sibling species of *An. gambiae* which transmit malaria worldwide have been described [9]. All of these vector-borne diseases occur mainly in tropical countries where more than two billion people live in endemic regions [10]. The approaches to combating vector-borne diseases relies on the interruption of the disease transmission cycle by either targeting the adult or larvae through spraying breeding sites or targeting adults using insecticides [11]. Synthetic pesticides have been extensively used for mosquito control by either killing the vectors, preventing adult mosquitoes from biting humans or by killing mosquito larvae at breeding sites [12, 13]. However, the development of resistance to different classes of synthetic insecticides such as pyrethroids, organophosphates, organochlorides and carbamates has drawn attention to the search of alternative methods of control [14]. The toxicity of the available chemical insecticides, their high operational cost and the subsequent environmental pollution have caused the need for developing alternative approaches to control vector-borne diseases [15]. Plant extracts with proven mosquito control potential can be used in place of synthetic insecticides, either as insecticides for killing larvae or adult mosquitoes or as repellents for protection against mosquito bites. Amongst them, essential oils have gained a special interest due to their insecticidal properties [16] andin recent years, have proved to be potentially useful sources of bioactive compounds against mosquito larvae [17]*.* Essential oils are composed of isoprenoid compounds, mainly monoterpene carriers of smell in the aromatic plants, such as sesquiterpenes alcohols identified from *Chamaecyparis obtusa* leaf oil and various monoterpene components derived from *Thuja orientalis* [18].

A number of studies have been conducted on essential oils to determine their larvicidal and repellency activities. *Cinnamon umzeylanicum*, *Cymbopogon citrates*, *Lavandula angustifolia*, *Tanacetum vulgare*, *Rubdosia melissoides*, *Eugenia caryophyllata*, *Ocimum* spp., *Gaultheria procumbens*, *Cuminum cymium*, *Bunium persicum*, *Trachyspermum ammi*, *Foeniculum vulgare*, *Abelmoschus moschatus*, *Cedru*s spp. and *Piper* spp. have been found to be effective for pest control [19]. Other essential oils such as lemon grass (*Cymbopogon winteriana*), eucalyptus (*Eucalyptus globules),* rosemary (*Rosemarinus officinalis*), vetiver (*Vetiveria zizanoides*), clove (*Eugenia caryophyllus*) and thyme (*Thymus vulgaris)* are known for their pest control properties [19]. Another study, conducted on the repellent effect of essential oils from *Cymbopogon* spp., *Ocimum* spp., and *Eucalyptus* spp. showed their strong repellency against malaria vectors and other mosquitoes [12].

Apart from essential oils, extracts prepared from Tanzanian plants have been evaluated for their larvicidal activity. For instance, extracts from *Annona muricata*, *Annona senegalensis* and *Annona squamosa* were active against *Culex quinquefasciatus*, which demonstrated that extracts of *Annona* species grown in Tanzania could be potential anti-mosquito agents [20]. In spite of the anti-mosquito activities of several studied species of plants, relatively little work has been done on the larvicidal activities of essential oils extracted from spices, such as clove and cinnamon. A recent study carried out in Nigeria assessed the activity of clove essential oils against *Ae. aegypti* and *Cx. quinquefasciatus* and achieved over 85% larval mortality within 24-hourspost-exposure [21]. Meanwhile, in Thailand, lemongrass and clove essential oils were effective in causing mortality for all larval stages of *An. dirus* and *Ae. aegypti* [22].

The aim of this study was to determine the active ingredients, and larvicidal activities of clove (Family: Myrtaceae) and cinnamon (Family: Lauraceae) essential oils and their blend against insectary-reared *Anopheles gambiae* (*s.s*.) larvae and *An. arabiensis* larvae from wild populations.

## Methods

### Collection of essential oils

Clove and cinnamon essential oils were purchased from Market Street in Mwanza, Tanzania. These oils were obtained raw through a distillation unit. There was no addition of any other contents.

### Larvae collection and rearing

Wild larvae of *An. gambiae * (*s.l*.) were collected from Magu district in Mwanza Region, North-Western Tanzania. Larvae were sampled from irrigated rice fields using a standard dipper (350 ml) [23] and transported to the insectary. Larvae were reared on yeast powder as a food source in the mosquito insectary unit at the Mwanza Research Centre, National Institute for Medical Research (NIMR), Tanzania. Colonies were maintained at 27 ± 2 °C, with a 12 h light: 12-h dark cycle.

Insectary reared third instars *An. gambiae * (*s.s*.) (Kisumu strain - insecticide susceptible strain) larvae and third-instar *An. arabiensis* field population larvae (insecticides tolerant population) were tested against essential oils in different concentrations. Larvae collected from wild population showed tolerance to pyrethroids and DDT in previous studies comprised of 91% *An. arabiensis* and the rest of *An. gambiae *(*s.s*.) and *An. funestus* [24]. The main source of insecticide resistance in this study site is the use of different insecticide classes with different mixtures in cultivating crops and the wide coverage of LLINs in the area [24].

### Mosquito larvicidal tests

Larvicidal tests were carried out based on the WHO standardized procedures and guidelines for larvicidal test method [25]. The initial screening was conducted using the selected plant extracts with different dosages ranging from 2 mg/ml to 100 mg/ml. The dosages causing mortality above 10% were selected for trial and ranged from 5 mg/ml to 80 mg/ml. Stock solutions of essential oils were prepared in acetone at an initial concentration of 160 mg in 2 ml (80 mg/ml). Then, two-fold dilutions were prepared in1ml acetone in each of the test cups to get a total of five different concentrations ranging from 80, 40, 20, 10, and 5 mg/ml of the essential oils.

Twenty third-instar larvae were placed in plastic cups containing distilled water for 1 h to reduce the stress. Thereafter 0.2 ml of each of the stock solution was added to their respective cups to make test solutions at concentrations ranging from 800, 400, 200, 100 and 50 μg/ml in their respective plastic cups containing the 20 test larvae each, which were used for the larvicidal tests. A negative control containing 19.8 ml of distilled water and 0.2 ml acetone with twenty third-instar mosquito larvae was also prepared. Each test comprised of four replicates of five concentrations (800, 400, 200, 100 and 50 μg/ml). Each sample of the essential oil was tested three times on different days in the four replicates. Acetone was used to solubilize the essential oil in the water. During the testing time, after 24 h of monitoring mosquito larvae, they were provided with yeast powder and monitored for 72 h.

Experiments were carried out under laboratory conditions 25–28 °C and a photoperiod of 12 h light followed by 12 h dark (12 L: 12D) against laboratory-reared third instar larvae of *Anopheles gambiae *(*s.s*.) and wild population of *An. arabiensis.* Mortality was recorded after 24 h of exposure, then larval food (yeast powder for *An. gambiae*) was added for those alive after 24 h, next mortality was recorded after 48 h and 72 h.

Larvae were considered dead when they were moribund or failed to do any movement. The dead larvae were counted, and the average percentage mortality was calculated. Data were adjusted for control mortality using Abbott’s formula when mortality in the control sets between 5% and 20% [26].

$$ Mortality\kern0.5em \left(\%\right)=\frac{X-Y}{X}\times 100 $$

where X was the percentage of survival in the control larvae population and Y was the percentage survival in the treated larvae population. The same formula was applied to both laboratory and wild larvae population.

Lethal dosages killing 50% (LC_50_) and 95% (LC_95_) of the population exposed were calculated using probit regression analysis in SPSS version 17.0 for windows (SPSS Inc., Chicago, IL, USA). A Student’s *t*-test was used to compare the mortality of larvae between concentrations for wild and laboratory-reared larvae in each evaluated extract and in blends.

### Chromatographic analysis

The identification of the essential oil components was carried out by high-resolution gas chromatography analysis, in the analytical laboratory, at Tropical Pesticides Research Institute (TPRI)-Arusha. The injection volume was 2 μl composed of 1.6 μl of a solution of essential oil (40 μg/ml) and 0.4 μl of a solution of hydrocarbon series of C_7_-C, as an internal standard, both in n-hexanes as a solvent. The gas chromatography coupled with mass spectrometry-GC-MS-system used consisted of a gas chromatograph, Thermo Scientific® Ultra GC coupled to a mass spectrometer, Thermo Scientific®**.** The fused silica capillary column used was a DB-5 J & W Scientific (30 m × 0.25 mm × 0.25 mm). Helium was the carrier gas, and the column temperature program was increased by 3 °C per minute between 60 and 240 °C. The mass spectra were obtained at 70 eV at a scan rate of 0.84 scans/s and at the range m/z 40–500. The retention times of sample components and a mixture of n-alkanes from C_7_-C, coinjected into the GC-MS system under the same temperature program was used for the calculation of the Arithmetic Retention Index - AI - and the Kovats Retention Index (KI) [27]. Identification of components was based on several methods: the calculated AI and KI and mass spectra [28].

## Results

**Fig. 1:**
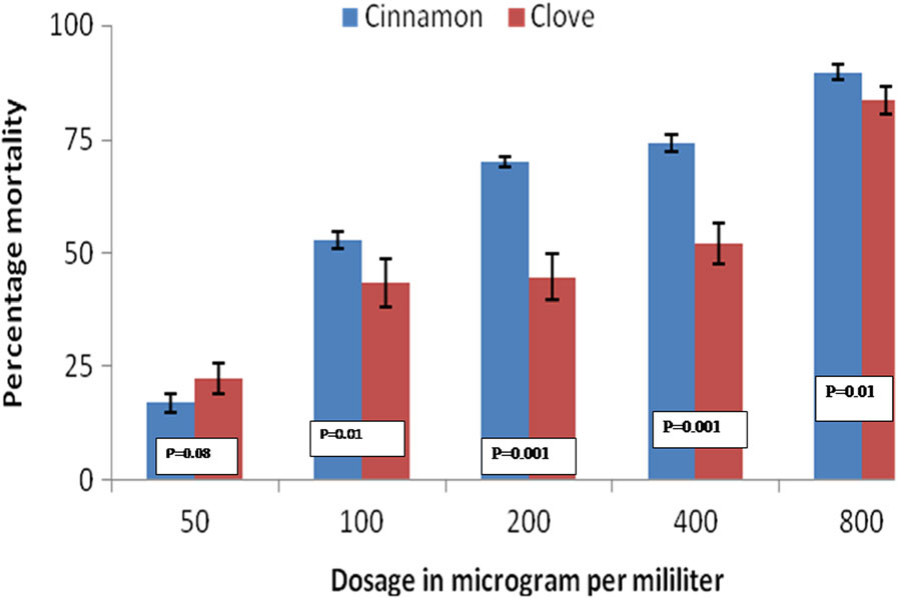
Larvae percentage mortality post-exposure in cinnamon and clove oils in different concentrations for wild-collected mosquitoes

**Fig. 2:**
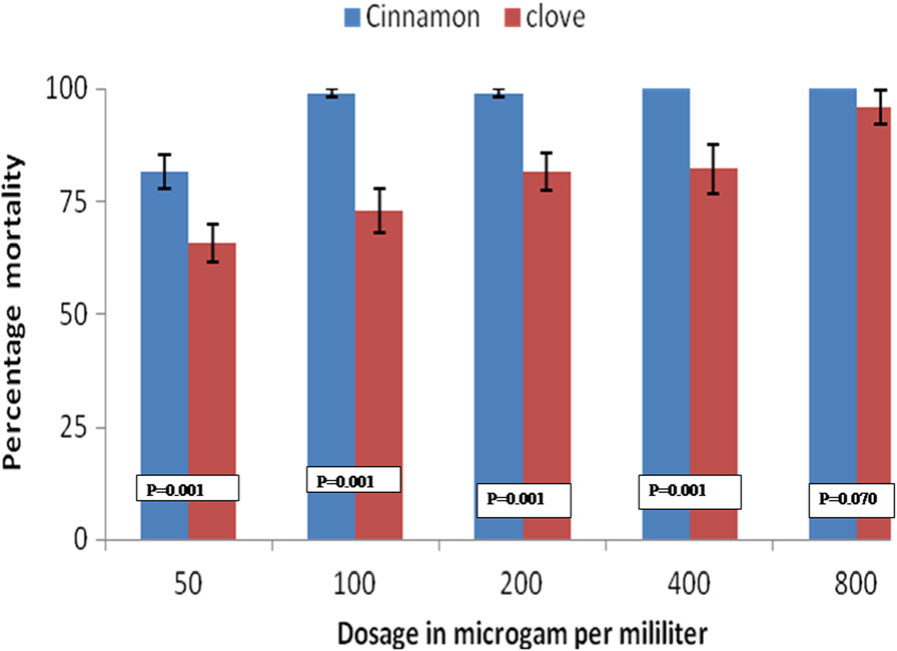
Larvae percentage mortality post-exposure in cinnamon and clove oils in different concentrations for laboratory-reared mosquitoes

**Fig. 3:**
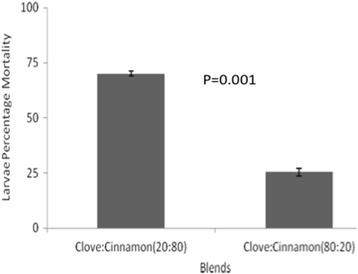
Percentage mortality of wild-collected larvae in two blends

**Fig. 4:**
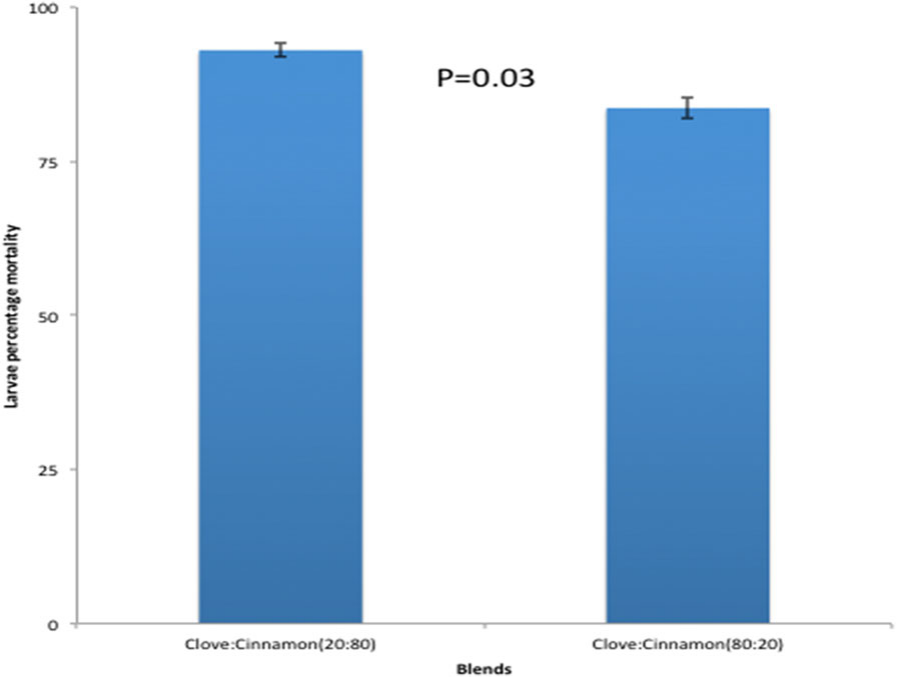
Percentage mortality of laboratory-reared larvae in two blends

A total of 2400 larvae were used for testing in different concentrations. For the laboratory-based susceptible colony, 1200 *An. gambiae *(*s.s*.) larvae were used, where 240 larvae in each of the five different concentrations were used. The similar wild population of *An.arabiensis* larvae collected from wild was tested in the same scenario as the laboratory-based population of *An. gambiae *(*s.s*.). The larvicidal activity of essential oils of cloves and cinnamon against laboratory and wild collected larvae were found to be dosage-dependent with a higher mortality observed for cinnamon essential oils (Figs. 1, 2). The difference in mortality between cinnamon and clove essential oils were statistically significant in each concentration for wild-collected and laboratory colonies except for the concentration of 800 μg/ml (Figs. 1, 2). The lethal dose to kill 50% and 95% of larvae population increased with the time of exposure for both laboratory-reared and wild population larvae (Tables 1, 2). The larvicidal activity of essential oil blends (of cloves and cinnamon*)* against *Anopheles gambiae *(*s.s*.) appeared to be higher in blends with higher proportions of cinnamon than clove essential oils (Figs. 3, 4). The chemical ingredients and composition of clove and cinnamon essential oils were found to be varying. The main active ingredient of cinnamon essential oils were Eugenol (96.5%) and β-Linalool (3.5%) (Table 3) while the main active ingredient of clove essential oils were eugenol (99.14%), phenol, 2-methoxy-4-(2-propenyl)-,acetate (0.45%), phenol, 2-methoxy-4-(1-propenyl)- (0.17%), methyl salicylate (0.13) and bicyclo [7.2.0]undec-4-ene, 4,11,11-trimethyl-8-methylene-,[1R-(1R*,4Z, 9S*)]- (0.12%) (Table 4).

**Table 1:** Mortality effect of clove and cinnamon essential oils against wild larvae with their LC_50_ and LC_95_ values

Essential oil	Time (h)	LC_50_(μg/ml)	95% CI	LC_95_(μg/ml)	95% CI
Clove	24	159.1	129.7–194.0	3950.1	2707.1–6311.2
	48	229.9	188.5–281.4	5707.6	3795.2–9488.8
	72	246.1	201.9–301.6	6110.8	4043.0–10,226.1
Cinnamon	24	131.459	119.8–143.9	1295.96	1128.0–1508.6
	48	129.668	118.2–141.9	1278.3	1113.5–1486.7
	72	115.536	105.1–126.7	1139.0	994.0–1321.6

**Table 2:** Mortality effect of clove and cinnamon essential oils against laboratory-reared larvae with their LC_50_ and LC_95_ values

Essential oil	Time (h)	LC_50 _(μg/ml)	95% CI	LC_95 _(μg/ml)	95% CI
Clove	24	17.527	1.38–48.29	2374.06	766.08–46,983.67
	48	19.56	1.68–52.42	2649.45	842.39–56,349.62
	72	8.605	0.34–29.24	1165.53	428.91–12,406.62
Cinnamon	24	11.788	5.67–18.87	115.15	86.85–158.69
	48	10.46	4.90–17.04	102.18	75.77–142.14
	72	12.314	6.07–19.43	120.28	90.97–166.96

**Table 3:** Chemical composition of the cinnamon essential oil

Compound	RT (min)	% composition
β-Linalool	5.666	3.5
Eugenol	10.483	96.5

*Abbreviation*: *RT* retention time in minutes

**Table 4:** Chemical composition of clove essential oil

Compound	RT (min)	% composition
Methyl salicylate	8.050	0.13
Bicyclo [7.2.0]undec-4-ene, 4,11,11-trimethyl-8-methylene-,[1R-(1R*,4Z, 9S*)]-	10.142	0.12
Eugenol	10.493	99.14
Phenol, 2-methoxy-4-(1-propenyl)-	11.281	0.17
Phenol, 2-methoxy-4-(2-propenyl)-, acetate	13.065	0.45

*Abbreviation*: *RT* retention time in minutes

## Discussion

Here, we showed the pronounced larvicidal activity of the investigated essential oils against wild larvae of *An.arabiensis* and laboratory-reared larvae of *An. gambiae *(*s.s.*) despite their different chemical components. Clove and cinnamon essential oils under investigation were found to have one common main active ingredient, Eugenol (4-alil-2-methoxyphenol). A similar chemical composition has been described for these essential oils with different extraction techniques [29]. The mortality caused by cinnamon was found to be higher than that caused by the clove essential oils, which might be attributed to the presence of β-linalool, found in cinnamon essential oils although in a small proportion. In a previous study, *Ocimum suave* essential oils extracts containing Eugenol as the highest occurring active ingredient had the lowest mortality in grain pests when tested in the absence of β-linalool than with β-linalool [30]. In previous reports, Eugenol mostly showed a high adult repellency effect rather than inducing larval mortality [31].

The mortality caused by clove essential oils was highly dosage-dependent with a higher mortality seen with the use of higher dosages. This trend was similar to another study conducted using the essential oils from *Terebinthifolia radii* [32]. In spite of being dosage-dependent, the mortality of the wild collected larvae was lower than that of laboratory-reared larvae. This may be explained by the fact that wild larvae have already been exposed to different insecticides and other environmental contaminants including heavy metals, which have caused them to be tolerant to different insecticides, unlike to the laboratory colonies [33, 34]. The mortality caused by the cinnamon essential oils had a similar trend as clove essential oils but higher mortalities, both in the laboratory and in the wild collected larvae, was observed with cinnamon essential oils.

The mortality of larvae caused by the two blends of cinnamon and clove in both the laboratory-reared and the wild larvae had a similar trend, with the blend having a higher proportion of cinnamon causing a higher mortality than the one with a higher proportion of clove. The high mortality with the increased proportion of cinnamon is explained by the presence of β-linalool in this essential oil. The mortality among susceptible laboratory larvae was higher than among wild-collected larvae due to the reported resistance gene dominance in wild populations [35]. This indicates that, if this active ingredient of cinnamon is appropriately synthesized and utilized for control purposes, it might have a great value in the existing vector control measures against insecticide tolerant vectors. In general, in agreement with the references quoted above, the effect of β-linalool component in cinnamon essential oil is definitely strongly associated with the mortality caused by this essential oil, either in the laboratory-reared or among the wild -collected larvae.

In spite of the great efficacy showed on mosquito larval mortality in different concentrations of tested plant-basedextracts, the main challenges remain the stability of the compounds in the natural environment. If collaboration with industries and chemists could be brought together, more stable compounds could be synthesized, such as to avoid the effect of sunlight, which causes degradation and form secondary metabolites which might be non-toxic and add no value in the fighting against malaria vectors.

## Conclusion

These essential oils of clove and cinnamon have shown to have a larvicidal effect which is concentration-dependent for both the laboratory-reared and the wild collected larvae. The proportion of the active ingredients in the cinnamon and clove essential oils blends have different mortality rateS among the laboratory-reared and the wild collected larvae. The extracted compounds can be useful if observed efficacy can be extended to small-scale trials with more synthetic compounds of similar structures.
